# Metasurface-driven polarization-division multiplexing of PCSEL for optical communications

**DOI:** 10.1186/s11671-023-03935-0

**Published:** 2023-12-07

**Authors:** Wen-Chien Miao, Chia-Hsun Chang, Fu-He Hsiao, Yun-Han Chang, Jhih-Hao Huang, Huan-Teng Su, Chang-Yi Lin, Chun-Liang Lin, Chi-Wai Chow, Yu-Heng Hong, Yao-Wei Huang, Hao-Chung Kuo

**Affiliations:** 1 Semiconductor Research Center, Hon Hai Research Institute, Taipei, 11492 Taiwan; 2 Department of Electrophysics, College of Science, National Yang Ming Chiao Tung University, Hsinchu, 30010 Taiwan; 3 Department of Photonics, College of Electrical and Computer Engineering, National Yang Ming Chiao Tung University, Hsinchu, 30010 Taiwan

**Keywords:** Metasurfaces, Photonic-crystal surface-emitting laser, Polarization-division multiplexing, Light communications

## Abstract

Free-space optical communications hold promising advantages, including a large bandwidth, access to license-free spectrum, high data rates, quick and simple deployment, low power consumption, and relaxed quality requirements. Nevertheless, key technical challenges remain, such as a higher transmission efficiency, a lower transmission loss, and a smaller form factor of optical systems. Here, we demonstrate the viability of circular-polarization-multiplexed multi-channel optical communication using metasurfaces alongside a photonic-crystal surface-emitting laser (PCSEL) light source at wavelength of 940 nm. Through the light manipulation with metasurface, we split the linearly polarized incidence into left and right circular polarizations with desired diffraction angles. Such orthogonal polarization states provide a paradigm of polarization division multiplexing technique for light communication. The PCSEL light source maintains a low divergence angle of about 0.373 degrees after passing through an ultra-thin metasurface without further bulky collimator or light guide, making end-to-end (E2E) and device-to-device (D2D) communications available in a compact form. Both light source and modulated polarized light exhibit a − 3 dB bandwidth over 500 MHz, with successful 1 Gbit/s transmission demonstrated in eye diagrams. Our results affirm that metasurface effectively boosts transmission capacity without compromising the light source's inherent properties. Future metasurface designs could expand channel capacity, and its integration with PCSEL monolithically holds promise for reducing interface losses, thereby enhancing efficiency.

## Introduction

Polarization plays a crucial role in understanding the wave nature of light, as it indicates the direction of light's vibrations. Various polarization states find extensive applications in diverse fields. For example, the polarization state can influence how light propagates through materials. When natural light encounters a polarizer, only the light aligned with the polarization direction can successfully pass through. This characteristic finds broad application in technologies like sunglasses, liquid crystal displays, and polarization imaging [1, 2]. Furthermore, through meticulous examination of the interplay between molecules and light manifesting distinct polarization orientations (e.g., circular polarization), a comprehensive investigation into intermolecular interactions can be conducted. This facet holds paramount significance in fields encompassing materials analysis and biological [3] and medical applications [4]. In the realm of optical communications and information technology, precise control over light polarization for the purpose of attaining information multiplexing stands to enhance the transmission rate and capacity of optical signals. This augmentation subsequently leads to the amelioration of communication system performance. It is noteworthy to mention that naturally occurring circularly polarized light is hardly observed in nature and can effectively mitigate interference when applied in the domain of communication. In 2014, Chi et al. [5] introduced the polarization division multiplexing technique (PDM), doubling the data transfer rate by utilizing an incoherent light-emitting diode (LED) while minimizing crosstalk with a pair of orthogonal linear polarizers. Later, Kwon et al. [6] further elevated the transmission rate from 1 Gbps to 2.04 Gbps by implementing PDM technology. In 2020, Chvojka et al. [7] effectively increase both data rate and spectral efficiency by 45% by employing PDM under orthogonal frequency division multiplexing (OFDM) signal transmission. Additionally, PDM technology has been synergistically integrated with multiple-input multiple-output (MIMO) [8] and/or wavelength division multiplexing (WDM) [9] technologies to further augment transmission capacity [10].

In recent years, dielectric metasurfaces have garnered significant attention due to their emerging nature, characterized by ultrathin structures, minimal absorption losses, and remarkable capabilities in manipulating light [11–13]. This novel, ultrathin, flat optical component has been applied across a broad array of fields, encompassing high numerical aperture lenses [14], waveplates [15], beam deflectors [16], polarization converters [17], beam shapers with holograms [18, 19], and polarization-tunable and orbital-angular-momentum-tunable lasers [20, 21]. Furthermore, metasurfaces are amenable to seamless integration with established semiconductor processes. This integration facilitates its direct incorporation into surface-emitting lasers, including vertical-cavity surface-emitting lasers (VCSELs) and photonic crystal surface-emitting lasers (PCSELs), enabling efficient manipulation of beam attributes and quality [22–27].

In today's optical communications market, direct-modulated high-speed lasers hold a dominant position. While distributed feedback (DFB) lasers and VCSELs are well-known, PCSELs stand out due to their exceptional features. These include a symmetric beam profile with narrow beam divergence and a narrower spectral width compared to VCSELs, making PCSELs promising contenders for next-generation light sources [28, 29]. Due to the ultra-small divergence angle (< 1°) of the PCSEL output beam profile, there is no need for collimating lenses or additional lens phase profiles in the optical system. Moreover, choosing near infrared (NIR) bands, which are imperceptible to the human eye, not only extends the optical communication bandwidth but also streamlines integration with other applications like security, bio-detection, and photodynamic/photothermal therapy [30, 31]. By utilizing GaAs for metasurface in the 940 nm band, it becomes feasible to directly etch nanostructures onto the substrate. This facilitates the seamless integration of metasurfaces with light-emitting elements. These characteristics contribute to a compact form, significantly reducing the form factor and optical complexity of the system.

In this article, we experimentally demonstrate a compact metasurface-PCSEL integration system that realize small form factor polarization-division multiplexing for light communications. We employ a 940 nm commercial PCSEL (L13395-04, Hamamatsu) as the illumination source, providing an ultra-small divergence angle as a lens-free configuration. The laser light beam from PCSEL directly irradiates onto the linear polarizer to provide a better linear polarization state. Then, the metasurface separates the incidence into left and right circular polarizations with designed diffraction angles. Such high efficiency and small divergence angle of orthogonal polarization beams from metasurface-PCSEL integration enhances transmission performance and thus provides a paradigm of PDM for light communication.

## Design and fabrication for meta-optical elements

To manipulate meticulous phase delay and light’s deflection direction coupling with circular polarization, we employ geometric phase, aka Pancharatnam-Berry phase [32] in our metasurface design. Rectangular nanopillars are common meta-atoms that construct a metasurface where phase delay of each meta-atom is simply defined by its rotation angle. Such strategy offers fabrication efficiency benefits by employing a consistent shape during the manufacturing procedure. The relation between phase delay and polarization of a meta-atom can be described by using Jones matrix. Within the framework of the electric field, in the linear polarization basis, they can be formulated as follows [33]:

1 $$\mathbf{M}=\mathbf{R}\left(-\alpha \right)\left(\begin{array}{cc}\widetilde{{t}_{L}}& 0\\ 0& \widetilde{{t}_{S}}\end{array}\right)\mathbf{R}\left(\alpha \right)$$

In the equation, $\widetilde{{t}_{L}}$ and $\widetilde{{t}_{S}}$ correspond to the complex transmission coefficients of a rectangular pillar along the long and short axes. The variable $\alpha$ signifies the orientation angle of the long axis, and R($\alpha$) represents the rotation matrix:

2 $$\mathbf{R}\left(\alpha \right)=\left(\begin{array}{cc}\mathrm{cos}\alpha & \mathrm{sin}\alpha \\ -\mathrm{sin}\alpha & \mathrm{cos}\alpha \end{array}\right)$$

Upon illuminating the meta-atom with incident LCP light, we acquire the following:

3 $$\mathbf{M}\frac{1}{\sqrt{2}}\left[\begin{array}{c}1\\ i\end{array}\right]= \frac{1}{2}\left(\widetilde{{t}_{L}}+\widetilde{{t}_{S}}\right)\frac{1}{\sqrt{2}}\left[\begin{array}{c}1\\ i\end{array}\right]+\frac{1}{2}{e}^{+2i\alpha }\left(\widetilde{{t}_{L}}-\widetilde{{t}_{S}}\right)\frac{1}{\sqrt{2}}\left[\begin{array}{c}1\\ -i\end{array}\right]$$

From Eq. (3), it is evident that upon illuminating the meta-atom with the LCP light, we can deconstruct the polarization state into a composite of LCP and RCP states. Simultaneously, by varying the rotation angle $\alpha ,$ a phase delay of 2 $\alpha$ can be achieved in the RCP state. If we opt for a condition where $\widetilde{{t}_{L}}+\widetilde{{t}_{S}}=0$ for the meta-atom, this signifies the potential for a full state conversion of LCP into RCP. Moreover, to quantitatively assess the capacity of polarization conversion for different and possible meta-atoms, the polarization conversion efficiency (PCE) can be estimated by the following formula [34]:

4 $$\mathrm{PCE}= \frac{{\left|\widetilde{{t}_{L}}-\widetilde{{t}_{S}}\right|}^{2}}{4}$$

**Fig. 1:**
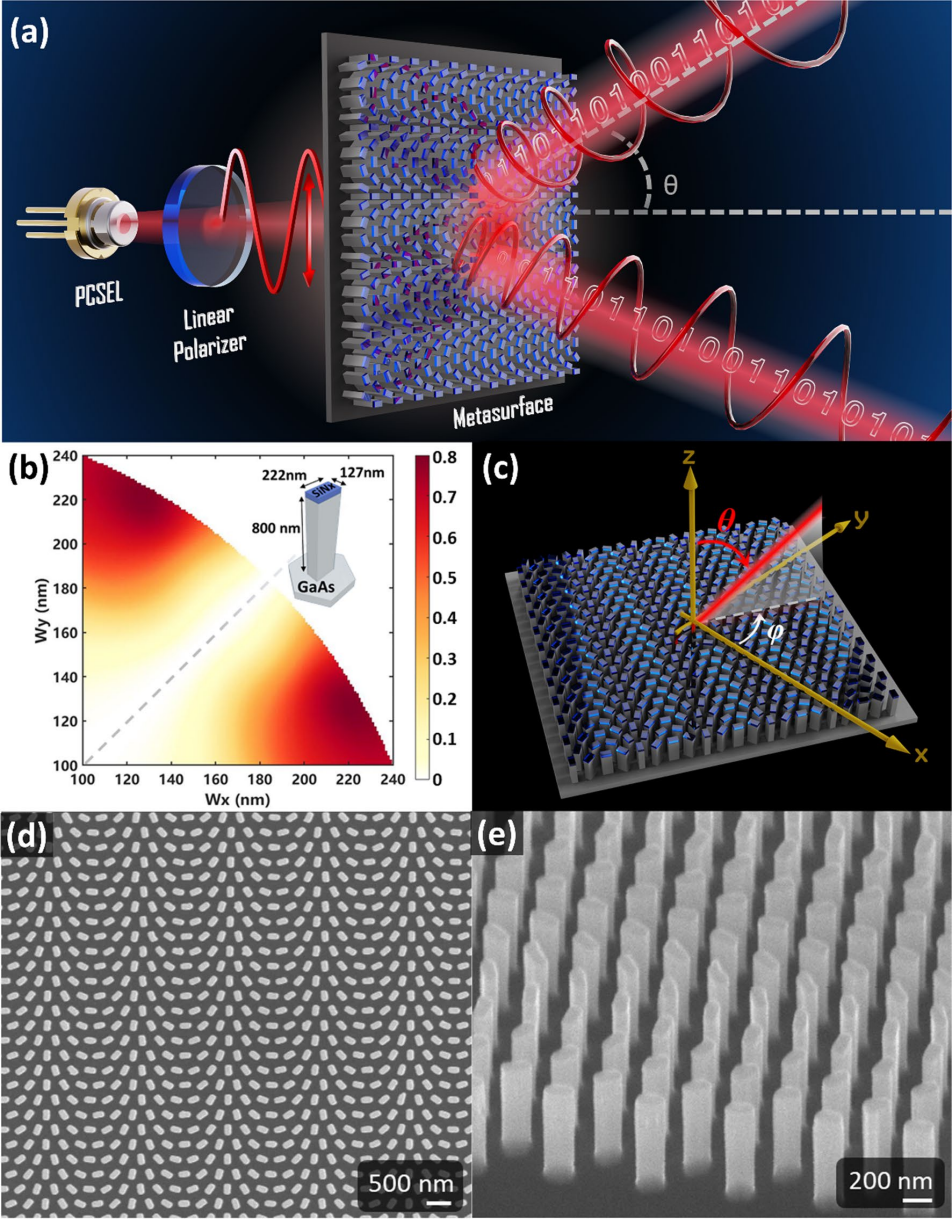
**a** Schematic shows a light communication system utilizing a metasurface and a PCSEL. Light beam from PCSEL irradiates onto the linear polarizer provides a better linear polarization state. The metasurface splits incidence into RCP and LCP beams with designed diffraction angles. **b** Polarization conversion efficiency (PCE) of different rectangle-shaped pillars with height of 800 nm height as building block for metasurfaces. The inset depicts an optimal configuration with the maximum PCE of 0.79, achieved by a pillar with the length of 222 nm and the width of 127 nm. **c** Schematic illustrates the polar angle and azimuthal angles with respect to the metasurface. **d** Top view and **e** side view SEM images of a fabricated metasurface

Drawing upon the theoretical framework and working wavelength of 940 nm, we employ gallium arsenide (GaAs) as the material for meta-atoms and adopt the hexagonal lattice arrangement with pillar-to-pillar distance (*U*) of 360 nm and high of 800 nm for higher conversion efficiency. Figure 1b illustrates the correlation between the length and width of meta-atoms and the PCE calculated with rigorous coupled-wave analysis (RCWA) method [35]. This observation is substantiated by simulation results indicating that the PCE exhibits symmetry along the diagonal (indicated by the gray dashed line), with the maximum conversion efficiency observed in structures characterized by a significant disparity between length and width. An optimal configuration with the maximum PCE of 0.79 can be achieved by a rectangle-shaped pillar with the length and the width of 222 nm and 127 nm, respectively. We note that the geometric parameters of the rectangular nanopillar and its rotation satisfy our fabrication feasibility, where there is a maximum aspect ratio of 8 for with and gap in our fabrication limitation. Following this, a series of geometric phase meta-atoms can be formed, employing the most efficient structure identified in Fig. 1b.

The ideal phase profile for beam deflection at desired angle is like a blazed grating, where the phase delay ($\varnothing$) of meta-atoms is described as follows:

5 $$\varnothing \left(x,y\right)=\frac{2\pi }{\lambda }\left(x\,\mathrm{sin}\theta\, \mathrm{cos}\varphi +y\,\mathrm{sin}\theta\, \mathrm{sin}\varphi \right),$$

where $\lambda$ is the operation wavelength, and $\theta$ and $\varphi$ are polar angle and azimuthal angle of diffraction beam in a spherical coordinate (Fig. 1c). We demonstrated three metasurface samples with polar angles of 15, 30, and 45 degrees while keeping azimuthal angle of 0 degrees in our experiment.

**Fig. 2:**
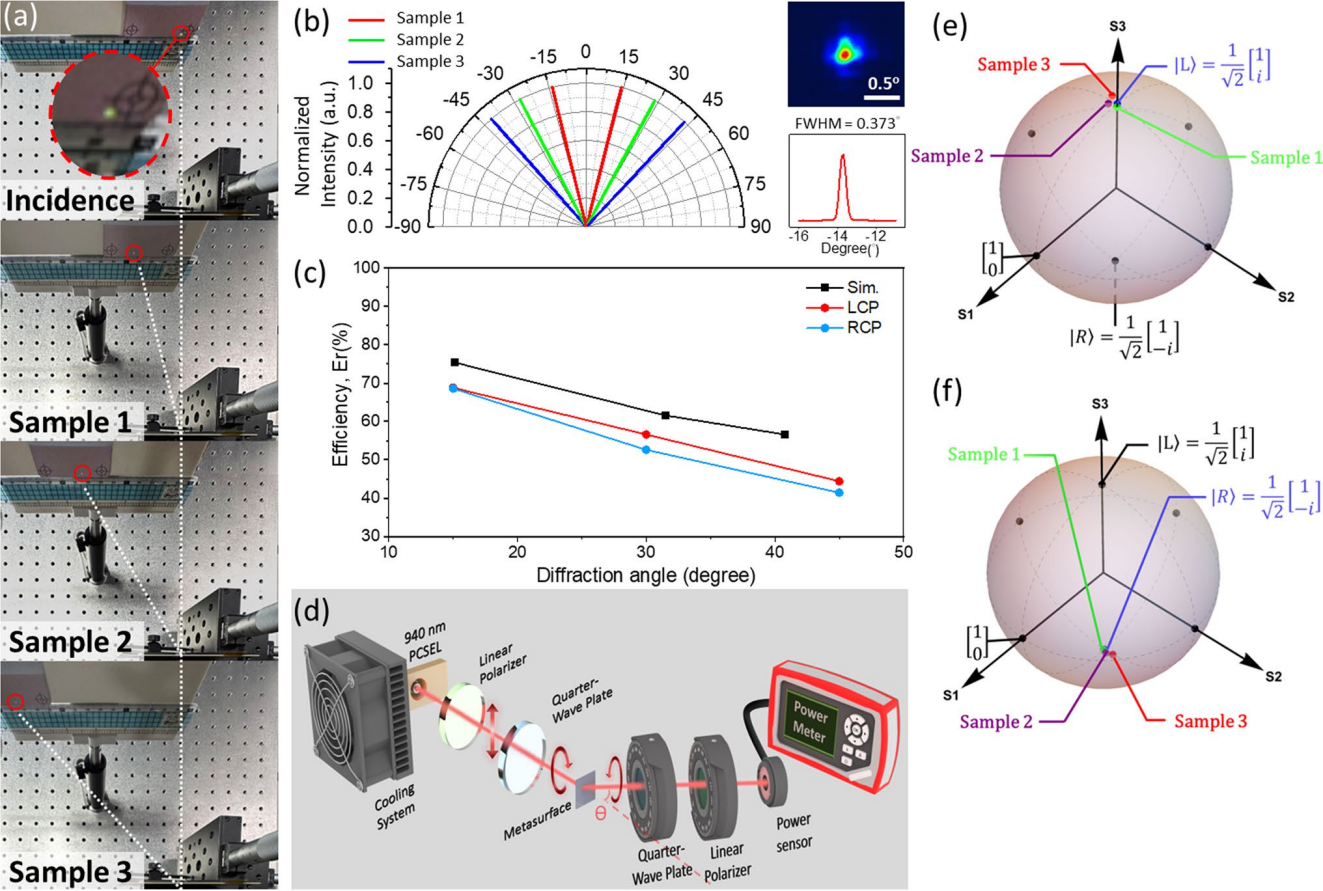
**a** Measured beam spots projected onto a far-field screen at *z* = 13.3 cm from metasurface samples in sequence. The diffraction angles of samples demonstrate their design at a range from − 15° to − 45°. The red circle and the inset: beam spot position on the screen. **b** Corresponding polar representation of the deflected beams. The inset shows the measured beam at diffraction angle of − 13.7°. **c** The simulated and measured efficiency of the metasurfaces with different diffraction angles. **d** Schematic shows the experimental setup for the degree of polarization. Herein, to facilitate an effective analysis of polarization states, the concepts of Poincaré sphere and the Stokes vector are employed, defining the degree of polarization. **e**, **f** The measured polarization state of diffraction beam at negative (**e**) and positive (**f**) diffraction angle, indicate LCP and RCP properties respectively

We realize our samples by using typical top-down fabrication. Initially, a 100 nm-thick Si_3_N_4_ layer is deposited onto a 360 μm-thick GaAs substrate, serving as a hard mask through plasma-enhanced chemical vapor deposition (Plasmalab80Plus, Oxford Instruments). Subsequently, a layer of negative electron-beam resist (ma-N 2403, Micro Resist Technology) is applied through spin-coating. The metasurface pattern is defined through electron beam lithography (VOYAGER, Raith), followed by the utilization of inductively coupled plasma (Plasmalab System 100, Oxford Instruments) for dry etching of Si_3_N_4_ layer. Eventually, the mixture of Ar_2_ and SiCl_4_ is employed to effectuate the pattern transfer from the Si_3_N_4_ hard mask onto the GaAs substrate. Finally, the scanning electron microscope (SEM, S-4700I, Hitachi) images of a sample are illustrated in Fig. 1c and d. The top view SEM image in Fig. 1c presents various rotation angles of rectangular pillars, in accordance with the desired grating phase profile corresponding to diffraction angle of 30°. Furthermore, the side view SEM image in Fig. 1d shows proficient GaAs etching techniques, where the nanopillar height measures approximately 800 nm, in line with expectations, and the edges appear to be nearly vertical. In addition, we also fabricate metasurface with diffraction angles of 15° and 45°, and we verify that the angles are consistent with the theory. Figure 2a illustrates the capability of a series of samples to different diffraction angles. Samples 1, 2, and 3 represent the metasurfaces with desired diffraction angle of 15, 30, and 45 degrees respectively. To gain a deeper understanding of their characteristics and performance, we conduct further analyses on their diffraction angles, divergence angle, efficiency, and degree of polarization (DOP).

Figure 2b depicts the beam deflection profiles of samples measured by using our homemade setup with angle resolution of 0.1°. The right deflection beams correspond to RCP converted from LCP component of incidence, and vice versa. The red color represents sample 1 with a 15-degree diffraction angle. The measured angles are located at 14.2° and − 13.7°, resulting in errors of − 5.3% and − 8.6%, respectively. The green color represents sample 2 with a 30-degree diffraction angle. The measured angles are 28.5° and − 27.6°, resulting in errors of − 5% and − 8%, respectively. The blue color represents sample 3 with a 45-degree diffraction angle. The measured angles are 43.1° and − 41.7°, resulting in errors of − 4.2% and − 7.3%, respectively. The lower right inset displays the magnified beam deflection profiles at − 13.7° from Fig. 2b, where the measured divergence angle (in full width at half maximum) is about 0.373°, indicating a slight increase of the divergence angle before passing through a metasurface (~ 0.300° [18]). The far field pattern measured by using beam profile meter (Beamage-4 M, Gentec-EO) shown in top right inset also matches the value. The small divergence angle from the metasurface-PCSEL integration indicates its collimation free and compact feature.

Furthermore, to estimate the efficiency of our metasurfaces, we adopt finite-difference time-domain method (FDTD, Lumerical) for the calculation. To mitigate the computational burden of the simulation, we streamline the model by incorporating periodic boundary conditions, focusing on the scenario when $\varphi$ equals 0. We assume the period length of the grating is $\Lambda$, which is *N* multipoles of pillar-to-pillar distance. Therefore, the simulated diffraction angle is:

6 $$\theta ={\mathrm{sin}}^{-1}\left(\frac{\lambda }{\Lambda }\right)={\mathrm{sin}}^{-1}\left(\frac{\lambda }{N\times U}\right)$$

The black line in Fig. 2c shows the simulated PCE up to 76.1% at diffraction angles of 15.13°, 31.48°, and 40.75°, corresponding to the *N* values of 10, 5, and 4 respectively. In experimental demonstration, we achieve diffraction angles of 15°, 30°, 45°, corresponding to the *N* values of 10.1, 5.22, and 3.69 respectively. The measured PCEs are shown in Fig. 2c as well, indicating a value up to 68.8% and decrease with larger diffraction angle as predicted in the simulation. Note that the *N* can only be designed as an integer because of the periodic boundary conditions in FDTD simulation but can be arbitrary number in experiment.

We measure the polarization state of diffraction beams by our homemade setup shown in Fig. 2d. A linear polarizer (LP, LPNIR100-MP2, Thorlabs) and a quarter-wave plate (QWP, 2-CPW-ZO-L4-0940, Laserand, Inc.) are located between the PCSEL and metasurface to generate an RCP or an LCP incident beam. There is another set of QWP and LP as an analyzer, accompanied with a power meter to measure the power of a beam projected on *x*-pol., *y-*pol., 45°-linear pol., 135°-linear pol., RCP, and LCP respectively. The measured Stokes vector of diffraction beams is calculated and listed in Table 1, where DOPs of the converted beams are near 1. Figure 2e and f present the Poincaré sphere of the diffraction beams at left and right angles, which are visualization of the rest Stoke parameters (S_1_, S_2_, and S_3_) listed in Table 1. The output states of samples 1–3 are close to the north and south poles of the sphere, indicating high purity of LCP and RCP output states and matching to our design.

**Table 1:** The normalized Stokes vector of PCSEL with different designed of metasurface

	Sample 1		Sample 2		Sample 3	
	RCP	LCP	RCP	LCP	RCP	LCP
S_1_	0.009	0.029	0.026	0.038	0.004	− 0.048
S_2_	− 0.002	0.020	0.038	− 0.061	0.105	− 0.096
S_3_	− 0.999	0.998	− 0.997	0.993	− 0.990	0.995
DoP	0.999	0.999	0.998	0.996	0.996	1.000

## Transmission properties

**Fig. 3:**
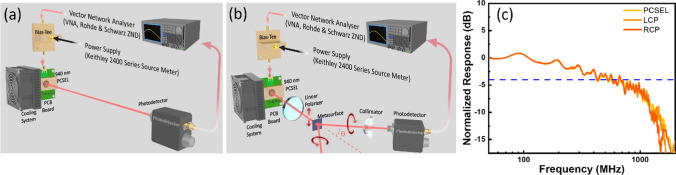
Schematic shows the experimental setup for optical response measurement. **a** without and **b** with metasurface; **c** the normalized frequency response for PCSEL with and without metasurface

Figure 3a and b illustrate the schematic depiction of the optical response measurement setup without and with metasurface. For the assessment of the optical response, a vector network analyzer (VNA, model: Rohde & Schwarz ZND) is employed during the experimental procedure. Alternating current signals generated by the VNA are integrated with a direct current signal (2400 Series Source Meter, Keithley) through the utilization of a bias tee. The PCSEL is affixed to the board through soldering and linked to the bias tee via optical fibers. To mitigate the risk of PCSEL overheating, which may potentially affect the results, the PCSEL is affixed to a copper heat sink and cooling system with thermal paste. This arrangement is designed to facilitate efficient heat dissipation and cooling. Moreover, a linear polarizer is positioned in front of the metasurface specimen to impose linear polarization upon the incident laser light. This linearly polarized light impinges upon the metasurface, subsequently generating both LCP and RCP light beams. The circularly polarized light signals are directed through a collimator and collected into a photodetector, which translates the optical signals into electronic signals suitable for input into the VNA. All the progression of light propagation, commencing from the laser source, traversing the polarizer and metasurface, and culminating at the light detector, are conducted within a free-space environment. Given the configuration of this experimental arrangement, the obtained bandwidth outcomes are depicted in Fig. 3c. The position of the − 3 dB bandwidth is indicated by the blue dashed line. The experimental findings indicate that when the PCSEL is driven at 300 mA, its − 3 dB bandwidth is about 560 MHz. The response curve exhibits oscillations between − 2 dB and − 5 dB within the 500 MHz to 1 GHz range and experiences a significant decrease beyond 1 GHz. The response characteristics of the metasurface-converted LCP and RCP light beams are similar to that of PCSEL. This result confirms that the conversion of polarization state through a metasurface does not affect the frequency response of light.

**Fig. 4:**
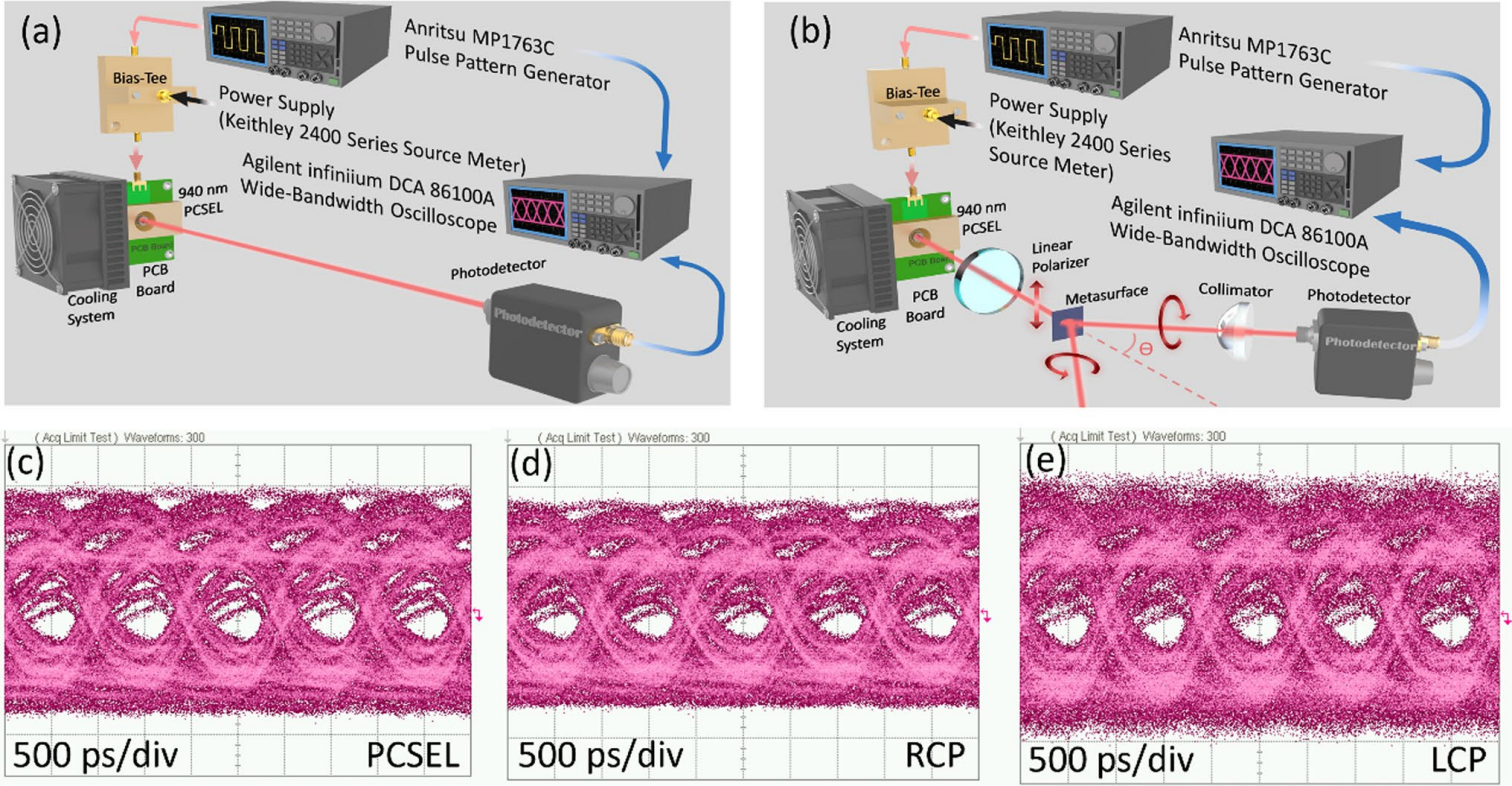
Schematic shows the experimental setup for the measurement of transmission performance **a** without and **b** with metasurface. The corresponding eye diagram at 1Gbit/s for **c** PCSEL without metasurface, **d** RCP and **e** LCP. The eye diagrams for both RCP and LCP are open and clear at 1Gbit/s, confirming the metasurface-driven polarization-division multiplexing of PCSEL for light communication

Figure 4a and b schematically depict the experimental setup for the measurement of transmission performance without and with the metasurface. The assessment of transmission performance for the PCSEL is carried out in the context of an On–Off Keying (OOK) system configuration. The investigated bit sequence comprised a Non-Return-to-Zero (NRZ) 2^7^ − 1 pseudorandom binary sequence (PRBS7), which is generated through a bit pattern generator (MP1763C, Anritsu). The corresponding analysis and recording of eye diagrams are conducted using a wide-bandwidth oscilloscope (Infiniium DCA 86100A, Agilent Technologies). The eye diagram of the PCSEL itself, as well as the LCP and RCP signals manipulated by the metasurface with a diffraction angle of 45°, are recorded with a transmission rate of 1 Gbit/s and an operational current of 300 mA. In all cases, clear and open eye diagrams can be observed at 1Gbit/s, as shown in Fig. 4c–e. Based on the outcomes derived from both bandwidth measurements and eye diagrams, both RCP and LCP signals exhibit comparable communication capabilities to that of the PCSEL itself, confirming the metasurface-driven PDM of PCSEL for light communication.

## Conclusion

In summary, we have demonstrated the feasibility of achieving polarization-multiplexed multi-channel optical communication using metasurface in conjunction with a commercially available PCSEL light source operating at around 940 nm. We successfully convert a single linearly polarized light beam into RCP and LCP light through metasurface manipulation, without affecting transmission characteristics or the divergence angle. Such orthogonal polarization states can provide a paradigm of PDM for light communication. Moreover, the PCSEL light source maintains a small divergence angle of approximately 0.373 degrees after metasurface manipulation as a lens-free configuration, making it suitable for end-to-end (E2E) or device-to-device (D2D) communications. Thus, through this polarization manipulation approach, we can enhance transmission performance within the realm of optical communication.

Both the light source and modulated polarized light exhibit a − 3 dB bandwidth exceeding 500 MHz, and eye diagrams indicated successful transmission at 1 Gbit/s. Our findings confirm that metasurface manipulation effectively enhances transmission capacity without compromising the light source's inherent transmission characteristics. The study shows the viability of achieving multi-channel optical communication transmission utilizing metasurfaces for PDM. Future designs of metasurface could increase the number of channels. With proper metasurface designs, the output generation of polarization states can be not only the typical linear and circular states but also vortex beams endowed with orbital angular momentum (OAM) [21]. This advancement not only bolsters transmission capacity in conjunction with polarization multiplexing [36] but also enables encrypted protection of signals [37], enhancing their security significantly. This technology holds immense potential for applications in underwater communication, Internet of Things (IoT), vehicle-to-vehicle communication, and low-earth-orbit (LEO) satellite technologies [38]. For practical usage, working efficiency of the metasurface in transmission is crucial. Several potential future perspectives have already been studied and explored by some pioneering research teams, such as introducing resonant modes with lower scattering loss [39, 40] and employing an inverse design approach to further improve the metasurface design [41, 42]. Moving forward, refining metasurface designs and their direct integration with PCSEL in a monolithic manner offer avenues to minimize interface loss and further enhance efficiency.

## Data Availability

The data presented in this study are available from the corresponding author upon reasonable request.
